# Application of Spontaneous Photon Emission in the Growth Ages and Varieties Screening of Fresh Chinese Herbal Medicines

**DOI:** 10.1155/2017/2058120

**Published:** 2017-01-31

**Authors:** Xiaolei Zhao, Jingxiang Pang, Jialei Fu, Meina Yang, Eduard Van Wijk, Yanli Liu, Hua Fan, Yufeng Zhang, Jinxiang Han

**Affiliations:** ^1^Department of Biochemistry and Molecular Biology, Shandong University, Jinan 250012, China; ^2^Shandong Medicinal Biotechnology Center, Key Laboratory for Biotech-Drugs of The Ministry of Health, Shandong Academy of Medical Sciences, Jinan 250062, China; ^3^Shandong Academy of Traditional Chinese Medicine, Jinan 250355, China; ^4^Sino-Dutch Centre for Preventive and Personalized Medicine/Centre for Photonics of Living Systems, Leiden University, Leiden, Netherlands; ^5^Meluna Research, Geldermalsen, Netherlands

## Abstract

Ultraweak photon emission emitted by all living organisms has been confirmed to be a noninvasive indicator for their physiological and pathological characteristics. In this study, we investigated the characteristics of spontaneous photon emission (SPE) and the contents of specific active compounds of roots and flowers buds of several fresh Chinese herbal medicines (natural medicines) with different growth ages and varieties. The results revealed that the contents of specific active compounds from same species herbs with different growth ages and varieties were significantly different, and this difference could be reflected by their SPE. Because the contents of specific bioactive constituents in Chinese herbs are closely related to their quality and curative effect, the SPE measurement technique may contribute to the quality control of Chinese herbal medicine in the future.

## 1. Introduction

All living organisms including animals, plants, algae, and bacterium spontaneously generate ultraweak photon emission (UPE), which is often called biophoton [1]. The emission ranges from the ultraviolet to near-infrared with intensities from tens to thousands photons per square centimeter per second [2]. The origin of this light energy is related to the chemiexcitation of biological molecules during oxidative metabolic processes [3, 4]. Therefore, the biophoton is closely related to various types of factors, such as exogenous toxins [5–7], pathogen attack [8, 9], temperature [10, 11], and acute stress [12–16], and it can reflect the physiological and pathological conditions of cells and tissues in a whole view. As a result, the measure of biophoton emission as a novel and noninvasive method has attracted considerable attention for monitoring the state of living organism and has been used in many fields, such as assessing cancer [17–20] and other health problems [21–26], detecting the quality of food [27], and the application in agriculture [7, 13].

Chinese herbal medicine (CHM; also known as Chinese herbs or Chinese materia medica), having a history of thousands of years, recently became widespread throughout the world due to their potential as an alternative complementary therapy and a source for discovering new drugs [28–30]. It possesses a holistic health care concept and regards disease as the imbalance of energy. Additionally, CHM adheres to the philosophy of cure instead of treatment [31]. The accumulated knowledge about CHMs makes their use safe and reasonable. However, the good and bad qualities of herbs have been intermingled in the market for the past few decades, which seriously affects the curative effect of Chinese medicinal formulae and the development of CHM. Therefore, how to discriminate the good qualities of herbs from bad ones becomes a thorny problem in the quality control of CHM [32]. There are many factors affecting the quality of herbs including growth ages, varieties, harvest season, processing, and storage [33]. However, it is hard to identify the varieties and growth ages and so forth of herbs only through their appearances, especially those of root herbs. It will seriously affect the quality of herbs from the source. In order to standardize the quality of CHM to some extent, a document named “General Guidelines for Methodologies on Research and Evaluation of Traditional Medicines” was enacted by World Health Organization in 2000 [34].

Since the active constituent(s) of the vast majority of herbs are often unknown, it is still inadequate to check the quality of herbs based on analyzing the constituents of them despite the fact that great efforts have been made in the quality control of CHM so far [31, 35, 36]. Therefore, the methods for quality control of CHM have not been recognized by the scientific communities currently. In order to discriminate the quality and identity of CHM meaningfully and properly, the new method retaining the traditional aspect of CHM needs to be employed. As the UPE reflects biological change in the holistic levels, which is consistent with the reflection of the CHM, the measurement of UPE is considered to be a promising means for the analysis of Chinese herbs. Recent studies have reported that DL were correlated significantly with the bioactive chemical constituents and can be used to predict and assess the Chinese herb's therapeutic properties [37, 38]. Taking into account the important roles of growth ages and varieties of herbs in their quality and the fundamental role of procurement quality of fresh herbs in the quality of CHM, additionally, verifying whether the spontaneous photon emission (SPE) could be used as a novel and noninvasive tool for the quality screening of fresh CHM, we carried out an investigation in the characteristics of SPE from the roots and flowers buds of several fresh CHMs considering their growth ages and varieties with the use of a sensitive photomultiplier tube (PMT) in this paper. And the contents of specific active compounds of these herbs were also analyzed, which could reflect the quality of herbs.

The results displayed that the contents of specific active compounds from same species herbs with different growth ages and varieties were significantly different, and this difference could be reflected by their SPE. These results indicated that this novel method may play a critical role in the quality screening of fresh CHMs in the future or at least in the screening of herbs with different growth ages and varieties

## 2. Materials and Methods

### 2.1. Spontaneous Photon Emission (SPE) Measurements

#### 2.1.1. SPE-Detection System

**Figure 1:**
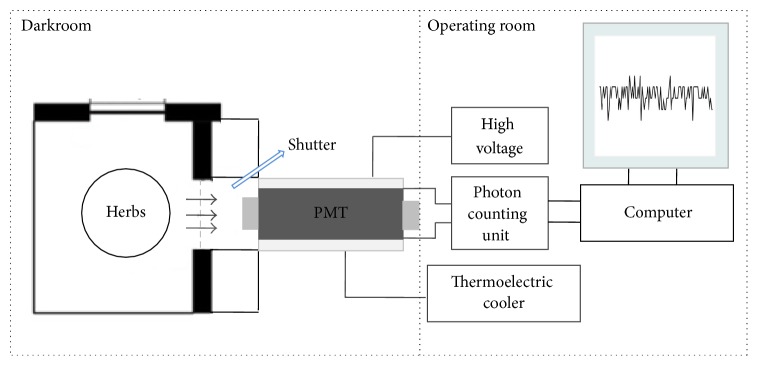
The schematic diagram of biophoton detection system used in this study.

Schematic diagram of the SPE-detection system used in our experimental study is shown in Figure 1. The components of this system include a dark chamber, a photomultiplier tube (PMT), a photon counting unit, a high voltage supply, a thermoelectric cooler, and a computer with photon count data software. The core components of the detection system are PMT R375 and photon counting unit C9744 (Hamamatsu Photonics K.K., Iwata, Japan). In front of the PMT is a shutter whose role is to avoid the disturbance of external light to the PMT.

The numbers of emitted photons from the samples were detected by the low noise and highly sensitive PMT working in single photon counting mode with a spectral response ranging from 160 to 850 nm and wavelength of maximum response was 420 nm. Subsequently, the photons were processed by the photon counting unit and fed into the computer which displayed the intensity in counts per 100 ms. To decrease the dark current (5 nA) and increase the sensitivity, the PMT was cooled down to −30°C using thermoelectric cooler C10372 (Hamamatsu Photonics K.K.), and the window of PMT was shielded by a dark chamber which was used to ensure light shielding and magnetic shielding. And then the PMT and the dark chamber were all placed in a dark room in order to eliminate the interference of external light further. In the dark chamber, a sample holder was set up not only to fasten the position of samples but also to ensure that the distance (3 cm) between the samples and the window of the PMT is same for all the measurement. Except for the PMT and the dark chamber, the other components of the detection system were placed in the operating room. During the period of measurement, temperature in the dark room was controlled at 25 ± 1°C by the temperature controller.

#### 2.1.2. Samples and Preparation

**Figure 2:**
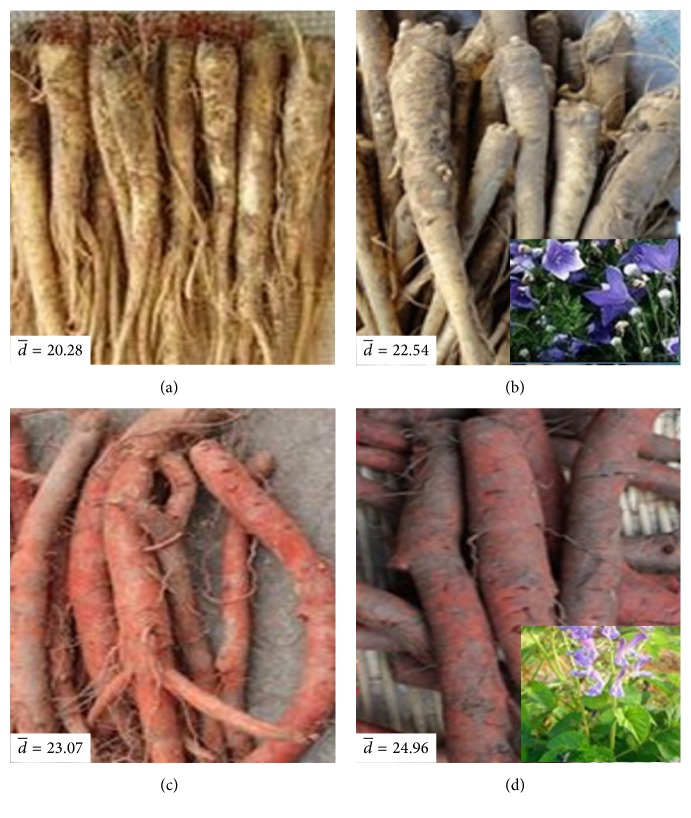
The roots of different growth ages of* purple flower P. grandiflorum* and* purple flower S. miltiorrhiza* in this study. (a) One-year-old* purple flower P. grandiflorum*; (b) two-year-old* purple flower P. grandiflorum*; (c) one-year-old* purple flower S. miltiorrhiza*; (d) two-year-old* purple flower S. miltiorrhiza*. $\overset{-}{d}$ means the average diameter of measured samples.

**Figure 3:**
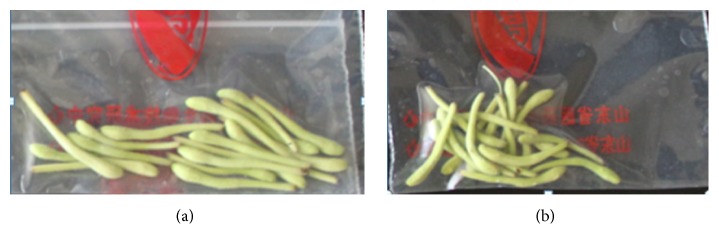
The flowers buds of different varieties of* L. japonica* used in this study. (a)* Four seasons of L. japonica*; (b)* Jiufeng number 1*.

**Figure 4:**
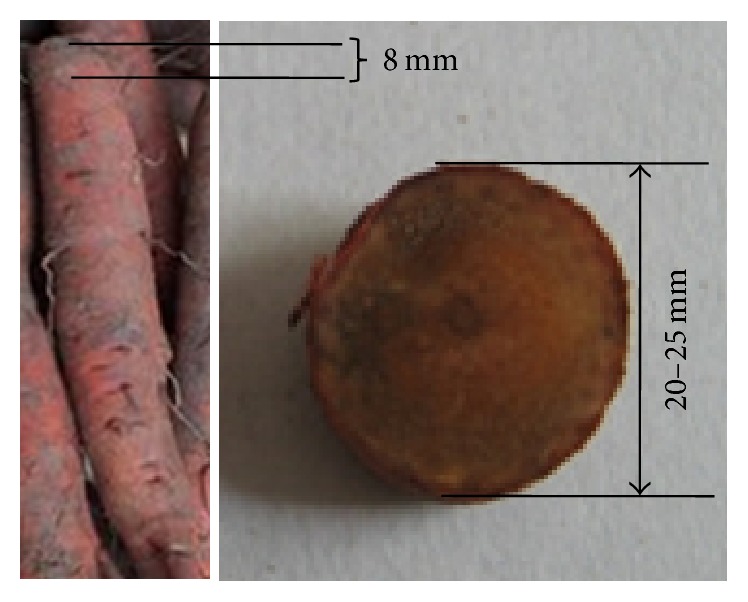
The diagram of slice sample of* S. miltiorrhiza*. The slice was cut from the top of the root and the thickness of all the slices was 8 mm. The diameter of all the slices ranges from 20 mm to 25 mm.

**Figure 5:**
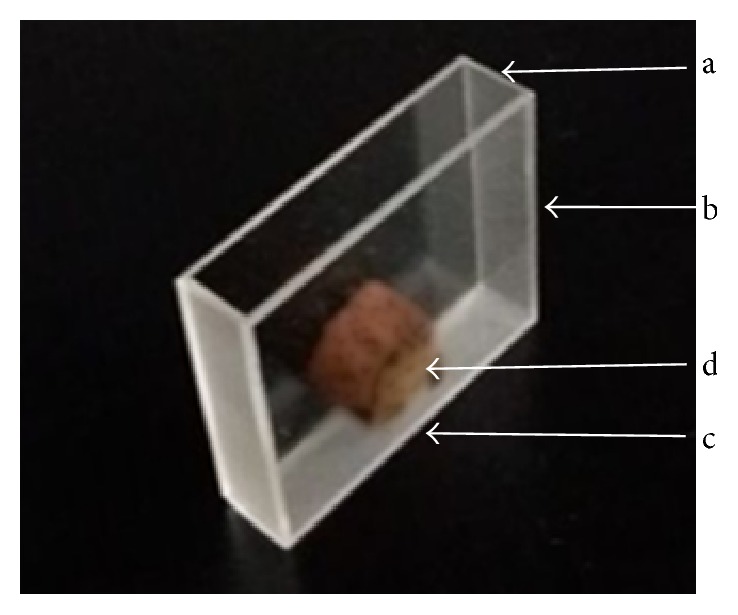
The diagram of cuvette used in this study. (a) The inside width of the cuvette was 1 cm. (b) The inside height of the cuvette was 4 cm. (c) The inside length of the cuvette was 5 cm. (d) The samples were placed in the middle of the bottom of the cuvette.

*P*.* grandiflorum*,* S. miltiorrhiza*, and* Lonicera japonica* (*L. japonica*) are planted in large areas in Shandong province. However, the growth ages and varieties of them are very confusing and they often mix together in the market. Unfortunately, some studies suggested that the growth ages and varieties of* P. grandiflorum* and* S. miltiorrhiza* seriously affected their active constituents, quality, and curative effect [35, 39–41]. Besides, there are more varieties of* L. japonica* in the market, which result in the error of clinical use of them and not reaching the curative effect that we expect [42]. Thus, the roots of different varieties of* P. grandiflorum* (*white flower P. grandiflorum* and* purple flower P. grandiflorum*) and* S. miltiorrhiza* (*white flower S. miltiorrhiza* and* purple flower S. miltiorrhiza*) with different ages (one-year-old and two-year-old) and the flowers buds of* L. japonica* (*four seasons of L. japonica* and* Jiufeng number 1*) were collected, respectively, as the experimental samples in this study. The photo samples were shown in Figures 2 and 3. Each kind of root samples with same growth age contained 30 samples collected from the same part of 30 plants in the similar growth states for 30 independent experiments in order to eliminate the effects by chance. And each variety of flower samples contained 120 flowers buds and was randomly divided into 20 portions (each portion includes 6 flowers buds) for 20 independent experiments. All of the samples were collected from the plantation of Shandong University of Traditional Chinese Medicine within about 1.5 h. At about 8:30 in everyday morning from 12 August to 30 August, the samples were collected. When the samples were picked, they were put in fresh-keeping bags and then preserved in a fresh-keeping box. Before measurement, each sample was pretreated as follows. For the roots of* P. grandiflorum* and* S. miltiorrhiza*, each sample was cleaned, one piece was cut from the top of each sample (the diagram of slice sample was displayed in Figure 4), and then the slices were put into clean quartz cuvettes (as shown in Figure 5(d)); for the flowers buds of* L. japonica*, each sample was cleaned and every 6 flowers buds as one portion were put into the clean quartz cuvette. Subsequently, their exposed areas represented by S to the PMT side were measured. In order to eliminate the influence of external light, the pretreated samples were placed in a completely dark room with controlled humidity (50%) and temperature (25 ± 1°C) for 0.5 h before measurement to eliminate delayed luminescence.

#### 2.1.3. Measurement Procedures

Each test always commenced at the same time of day (10:30 a.m.) in order to try to keep all of the measurement conditions consistent. The procedures for measuring SPE were as follows. The background emission (BG) of the blank cuvette without any sample was measured for 3 min. Then, the shutter was closed and the processed samples were put inside the dark chamber on the sample holder of the detection system under the condition of complete darkness within 30 s. After that, the shutter was opened and the SPEs of the samples were recorded. We sequentially measured 10 samples each day and each sample was measured 3 times. In order to shorten the total measurement time, to keep the samples fresh, we set the duration of each measurement for 5 min. In the whole measurement process, the time interval (Δ*t*) was 100 ms. The obtained data were finally corrected by the corresponding background emissions.

### 2.2. Determination of Specific Bioactive Constituents of Chinese Herbs

The contents of specific bioactive constituents in Chinese herbs are closely related to their quality and curative effect. Therefore, we sent our herbal samples to the Shandong Institute for Food and Drug Control for detecting the specific bioactive constituents in order to analyze whether the contents of specific active compounds of herbs with different growth ages and varieties were different. Total saponins in* P. grandiflorum*, tanshinone IIA, and salvianolic acid B in* S. miltiorrhiza*, galuteolin, and chlorogenic acid in* L. japonica* were detected, respectively, according to the Chinese Pharmacopoeia and relevant literature.

### 2.3. Data Analysis

All of the data of SPEs from each sample were normalized by their exposed areas. And the statistical analysis of photon count data of samples was presented in mean ± SD (standard deviation) which was performed with SPSS 18.0 software. The data graph was made with program Origin 8.0. The paired *t*-test was used to compare the data from the samples with two different growth ages and varieties. In all statistical tests, *P* ≤ 0.05 was considered significant.

## 3. Results

### 3.1. The Differences of SPE Intensity and Contents of Specific Bioactive Constituents between the Samples Differing in Growth Age

**Figure 6:**
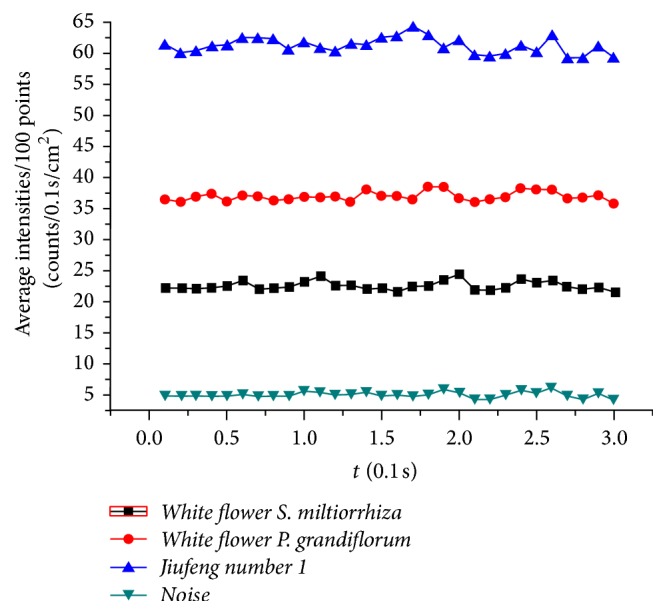
The average intensity of every 100 points of SPE from the roots of* white flower P. grandiflorum* (red dot) and* white flower S. miltiorrhiza* (black square) with two years of age as well as the* L. japonica*'s flowers buds of* Jiufeng number 1* (blue equilateral triangle) and the background noise (dark green inverted triangle).

**Figure 7:**
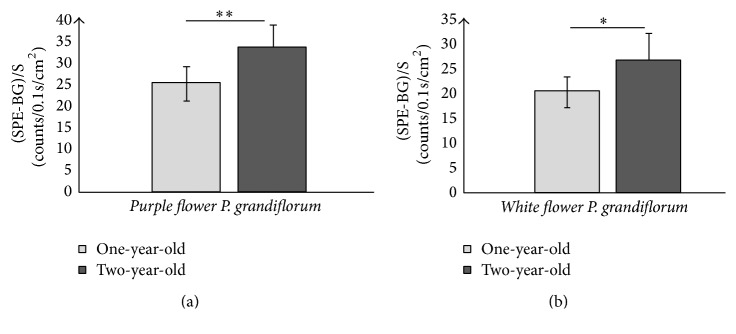
Comparisons of SPEs between different growth ages of roots of* purple flower P. grandiflorum* (a) and* white flower P. grandiflorum* (b). ^*∗*^*P* < 0.05; ^*∗∗*^*P* < 0.01. *P* values were obtained by paired *t*-test.

**Figure 8:**
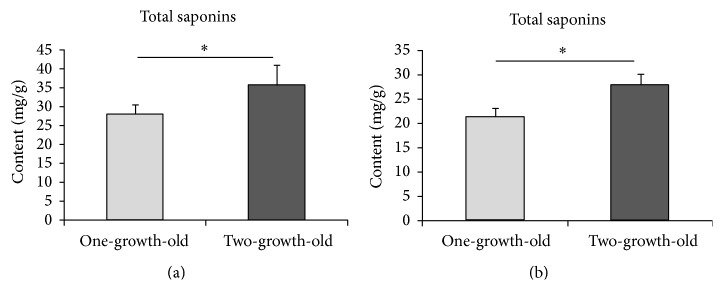
Comparisons of contents of total saponins between different growth ages of roots of* purple flower P. grandiflorum* (a) and* white flower P. grandiflorum* (b). ^*∗*^*P* < 0.05 (two-tailed, paired *t*-test).

**Figure 9:**
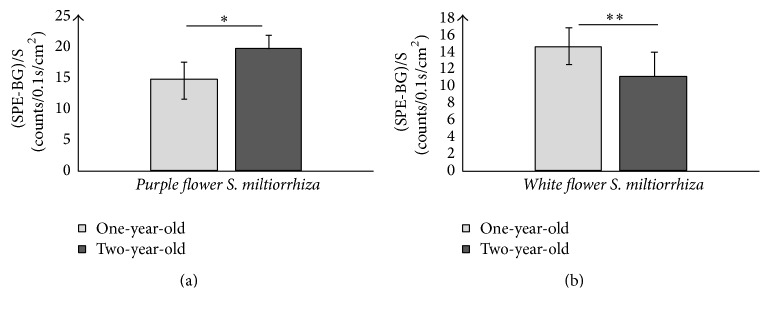
Comparisons of SPEs between different growth ages of roots of* purple flower S. miltiorrhiza* (a) and* white flower S. miltiorrhiza* (b). ^*∗*^*P* < 0.05; ^*∗∗*^*P* < 0.01. *P* values were obtained by paired *t*-test.

**Figure 10:**
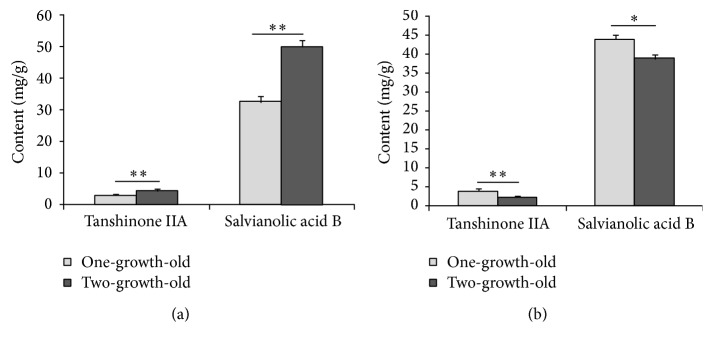
Comparisons of contents of tanshinone IIA and salvianolic acid B between different growth ages of roots of* purple flower S. miltiorrhiza* (a) and* white flower S. miltiorrhiza* (b). ^*∗*^*P* < 0.05; ^*∗∗*^*P* < 0.01. *P* values were obtained by paired *t*-test.

The SPE and contents of specific bioactive constituents of roots of* white flower P. grandiflorum*,* purple flower P. grandiflorum*,* white flower S. miltiorrhiza*, and* purple flower S. miltiorrhiza* with different growth ages (one-year-old and two-year-old) were measured, respectively, for analyzing the difference of SPE and contents of specific bioactive constituents between the different growth ages of herbs.  Figure 6 displayed the intensity of SPEs in time from the roots of* white flower P. grandiflorum* (red line) and* white flower S. miltiorrhiza* (black line) with two years of age as well as the* L. japonica*'s flowers buds of* Jiufeng number 1* before removing the background (BG), which illustrated that the influence of the external light source on the samples has been eliminated. And Figures 7–10 showed the average SPE intensity (after the subtraction of BG) and average contents of specific bioactive constituents of samples with different growth ages. The total BGs were measured as 5 ± 1 counts/0.1 s, and the significant discrepancies between the BGs and all the samples were observed (*P* < 0.001).

As shown in Figure 7, the SPEs intensities of two-year-old* P. grandiflorum* were significantly higher than those of the one-year-old samples in two species (*P* < 0.05). And the contents of total saponins which associated with the quality of* P. grandiflorum* shown in Figure 8 illustrated that one-year-old* P. grandiflorum* only contained less than 65% of total saponins of two-year-old one and the contents of total saponins of one-year-old* P. grandiflorum* were about 20% lower than the provisions of “Chinese Pharmacopoeia.” Therefore, one-year-old* P. grandiflorum* was labeled bad quality herbs.  Figure 9 displayed that the SPE intensity of two-year-old* purple flower S. miltiorrhiza* was significantly higher than that of the one-year-old sample; however, this result was contrary to the* white flower S. miltiorrhiza*: the SPE intensity of two-year-old* white flower S. miltiorrhiza* was lower than that of the one-year-old sample. Interestingly, the data in Figure 10 showed that the contents of tanshinone IIA and salvianolic acid B in two-year-old* purple flower S. miltiorrhiza* were significantly higher than those of one-year-old sample, while the contents of those two active constituents in two-year-old* white flower S. miltiorrhiza* were lower than those of one-year-old sample. These results indicated that the growth age of herbal roots could affect their SPE intensity and there was a close relationship between the bioactive constituents of fresh herbs and their SPE intensities. Besides, the high deviation of SPE intensities among samples suggested that SPE was extremely sensitive and varied in a wide range for slight difference in different plants.

### 3.2. The Difference of SPEs and Contents of Specific Bioactive Constituents of the Samples in Different Varieties

**Figure 11:**
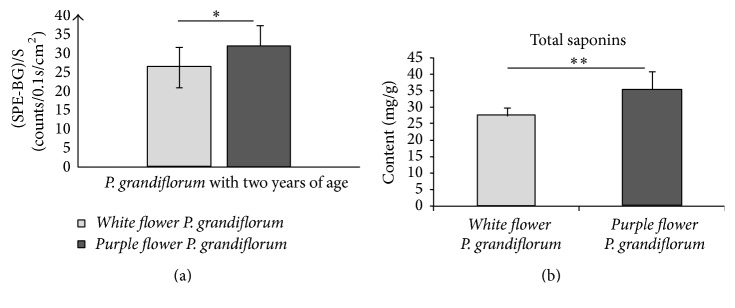
Comparisons of SPEs (a) and contents of total saponins (b) between different varieties of two-year-old roots of* P. grandiflorum*. ^*∗*^*P* < 0.05; ^*∗∗*^*P* < 0.01. *P* values were obtained by paired *t*-test.

**Figure 12:**
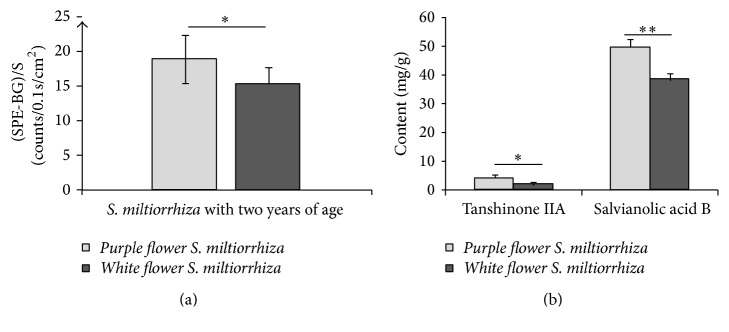
Comparisons of SPEs (a) and contents of tanshinone IIA and salvianolic acid B (b) between different varieties of two-year-old roots of* S. miltiorrhiza*. ^*∗*^*P* < 0.05; ^*∗∗*^*P* < 0.01. *P* values were obtained by paired *t*-test.

The data of SPE from the roots with different growth ages of* P. grandiflorum* and* S. miltiorrhiza* revealed that some interesting difference of SPE existed in different varieties. The data of (SPE-BG)/S from the two-year-old roots of different varieties of* P. grandiflorum* and* S. miltiorrhiza* were obtained by averaging 30 independent experiments, and then the contents of specific bioactive constituents of different samples were detected. The results were displayed in Figures 11 and 12.

**Figure 13:**
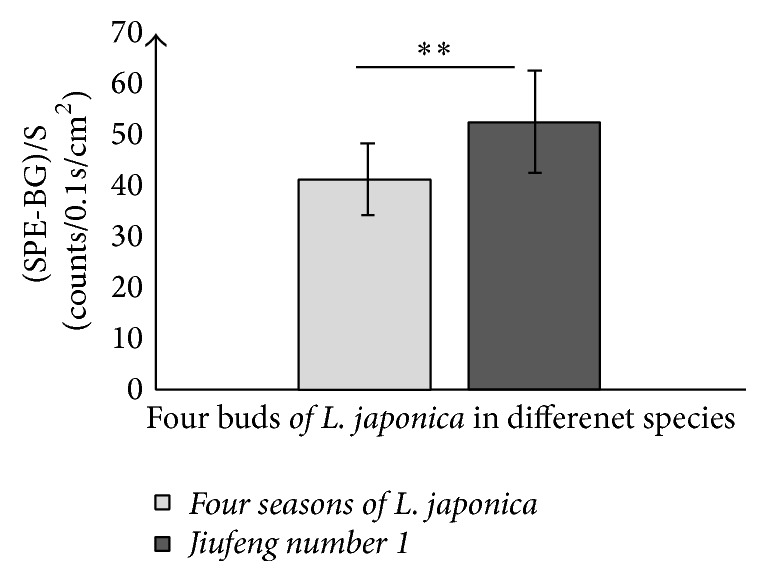
Comparisons of SPEs between different varieties of flowers buds for* L. japonica*. ^*∗∗*^*P* < 0.01. *P* values were obtained by paired *t*-test.

**Figure 14:**
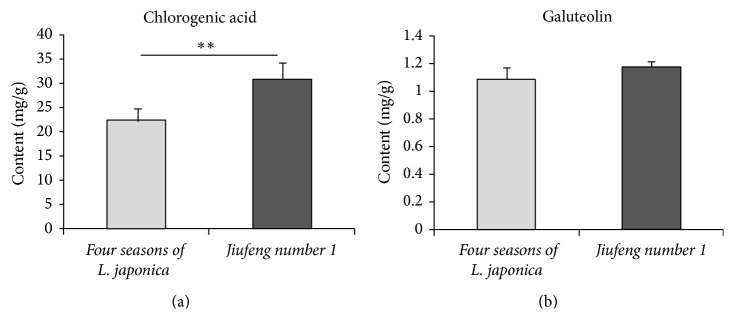
Comparisons of the contents of chlorogenic acid (a) and galuteolin (b) between different varieties of flowers buds for* L. japonica*. ^*∗∗*^*P* < 0.01. *P* values were obtained by paired *t*-test.

The results suggested that SPEs intensities and contents of specific bioactive constituents from two-year-old roots of different plants varieties showed significant differences (*P* < 0.05). And the change tendencies of SPE intensity and the contents of specific bioactive constituents between different varieties of* P. grandiflorum* and* S. miltiorrhiza* were the same. These results indicated that the contents of specific active compounds from different varieties herbs were different and there was a close relation between the varieties of plants and their SPE. In order to demonstrate this phenomenon further, the SPEs and the contents of galuteolin and chlorogenic acid of flowers buds from different varieties of* L. japonica* (*four seasons of L. japonica* and* Jiufeng number 1* as shown in Figure 3) were measured with the measurement procedures. Subsequently, the paired *t*-test was made and the SPE intensities of 20-portion samples in both varieties were averaged in mean ± SD. The outcomes were displayed in Figures 13 and 14. Notably, all of the flowers buds were in the same growth states.

The result in Figure 13 exhibited that the average intensity of SPE from* Jiufeng number 1* was higher than that from* four seasons of L. japonica*. In Figure 14, the content of chlorogenic acid in* Jiufeng number 1* was significantly higher than that from the* four seasons of L. japonica*, though the content of galuteolin in these two varieties of* L. japonica* was the same. This result confirmed again that the herbal varieties could affect their SPE intensity and there was a close relation between the bioactive constituents of fresh herbs and their SPE.

## 4. Discussion

SPE is an intrinsic property of biological systems. Present studies have suggested that it originates from the relaxation of electronically excited varieties formed in the biological systems during the normal or abnormal oxidative metabolic process [43, 44]. The production of reactive oxygen varieties (ROS: O_2_^•−^, HO^∙^, H_2_O_2_, ^1^O_2_) in metabolic process plays a key role in the photon emission [45, 46]. In plants, generation of ROS is related to the enzymatic reactions in mitochondria, chloroplast, and cytoplasm, and the cellular respiration in mitochondria is a main source [42, 47]. It is stated that ROS with a high positive redox potential has an bility to oxidize various cellular biomolecules such as lipids, proteins and nucleic acids, and then it initiates cascade reactions which are accompanied with the generation of electronically excited varieties such as triplet excited carbonyls (^3^R=O^*∗*^), excited pigment (*P*^*∗*^), and singlet oxygen (^1^O_2_), resulting in photons emission [4, 43, 48–50]. Thus, the intensity of photon emission is the reflection of underlying metabolic processes in cells.

It is known that the oxidative metabolic processes are the fundamental chemical reactions in the biological system and they reflect the physiological conditions of their system. Therefore, the applications of SPE for the detection of metabolic processes in biological system are widely used. Present studies have suggested that the biophoton emission is a meaningful indicator of physiological state in biological systems [8–16, 16–20, 51–53]. In this view, we measured the SPE and contents of specific active compounds of roots and flowers buds with different growth ages and varieties of several fresh Chinese herbal medicines in order to prove whether the growth ages and varieties of fresh herbs could affect their SPE intensity and whether there was a close relationship between the bioactive constituents of fresh herbs and their SPE intensities.

Our data showed that the herbs with different growth age and variety displayed different SPE intensity and content of specific active compounds. In addition, the SPE intensity might have some relationship with the content of specific active compounds of herbs. This may be because the number and types of genes and proteins in different growth ages and varieties of plants are different from each other and these result in varying concentration of specific active compounds. As a consequence, the rate and type of metabolism will also be different, leading to the difference of SPE. Hence, the SPE of a fresh herb is related to its growth age and variety and it was partially correlated to the content of specific active compounds in it at the same time, which reflects the quality and curative effect of herbs. However, further validation is required. Thus, by further research, the SPE of fresh herbs may be a useful index to reflect their growth ages and varieties noninvasively in the future.

It is worth noting that, before we apply this technique practically, we need a large amount of systematic and standard detection for the herbs with different growth ages and varieties using the SPE detection technique. Firstly, these herbs are collected from the field in the way of random sampling and identified by the experts. And then a mutilevel database was found corresponding to the relationship between the various growth ages/varieties of herbs and the characteristics of SPE. Subsequently, the database was optimised according to the actual need. Finally, a feasible “standard” was set up. Once the “standard” was set up, the advantages of the SPE technique can be sufficiently exerted

## 5. Conclusion

In this paper, we reported the changes of SPE intensities and contents of specific active compounds along with growth ages and varieties of several fresh Chinese herbal medicines. Our data showed the statistically significant differences in the photon emission intensities and the contents of specific active compounds between different growth ages and between different varieties of herbs. Although the precise mechanism of SPE has not been fully clarified, these results indicated that the contents of specific active compounds of fresh herbs with different growth ages and varieties were significantly different, and this difference could be reflected by their SPE. Therefore, the SPE may be an effective indicator for discriminating herbs with different growth ages and different varieties which greatly affect the quality of Chinese herbs. In this sense, by continuous measurement of SPE from many more types of herbs with different growth ages, varieties, harvest seasons, authenticity, and so forth, this novel and noninvasive method may make a great contribution to solving the tough problems existing in the quality screening of fresh Chinese herbal medicine in certain degree in the future.
